# Editorial: Patient focused developments in food allergy

**DOI:** 10.3389/falgy.2023.1287078

**Published:** 2023-09-26

**Authors:** Jonathan S. Tam, Neema Izadi, Joyce E. Yu

**Affiliations:** ^1^ Division of Clinical Immunology and Allergy, Children’s Hospital of Los Angeles, Los Angeles, CA, United States; ^2^ Division of Allergy, Immunology, and Rheumatology, Department of Pediatrics, Columbia University Irving Medical Center, New York, NY, United States

**Keywords:** food allergy, patient, consequence, shared decision making, prevention

**Editorial on the Research Topic** Patient focused developments in food allergy

Food allergy is one of the most common chronic conditions of childhood and affects up to 10% of children (1). Perhaps because of its increase in prevalence over the past 30 years, it is not surprising that there has been a significant growth in academic research and awareness from the general public. Without a doubt, great strides have been made in not only understanding the immunology of food allergy but also in potential therapeutic options for patients. While at this time there is only one FDA approved therapeutic for peanut allergy, a number of immunotherapeutic approaches are currently under investigation—including different routes of immunotherapy (oral, sublingual and epicutaneous), immunotherapy with modified recombinant proteins, and use of biologics (with and without immunotherapy) (2).

However, despite these advances, the general experience for the vast majority of patients has not changed. Fundamentally, real change in understanding of food allergy, treatments and diagnostics have had little impact on clinical practice. Moreover, despite the best intentions, efforts directed at affecting change have historically not always led to the best results.

Prevention of food allergy has long been a target in the effort to stem the rising tide of food allergy. Over 20 years ago, a guideline by the American Academy of Pediatrics (published in 2000) recommended the use of hydrolyzed formulas and delayed introduction of allergenic foods including peanut until the age of 3 years. The guidelines were based on expert opinion at the time and on studies from the preceding 2 decades which seemed to support the recommendation. However, the prevalence of food allergies continued to increase and in 2008 the guidelines changed to remove those specific recommendations. The guidelines would completely change again following the publication of the results of the Learning Early About Peanut (LEAP) trial (3). Feeding guidelines across the globe changed dramatically promptly encouraging the early introduction of allergenic foods, particularly in high-risk infants (4). While the full impact of the change to early introduction is still being evaluated, recent results highlight two important lessons on the implementation of promising research—generalizability and unintended consequences. A report from Australia (5) looking at 2 population-based cross-sectional samples used to evaluate the prevalence of peanut allergy before and after introduction of Australia's new infant feeding guidelines revealed minimal effect on peanut allergy prevalence; thus, underscoring the potential difficulty in translating research to a population.

In terms of unintended consequences, the quest to reduce immediate food allergic reaction to peanuts may have unintentionally increased a different type of food allergy—food protein induced enterocolitis syndrome (FPIES). Shortly after the establishment of the new guidelines, a series of three cases of FPIES to peanut were reported (6). Subsequently, a retrospective analysis identified 14 cases of peanut FPIES from 2019 to 2021 in stark contrast to a previous review of 160 patients with FPIES in the same institution from 2001 to 2011, with no reported cases of peanut FPIES (7).

Even efforts which may seem innocuous or generally benign can have strange consequences. In the United States, the Food Allergy Safety, Treatment, Education and Research (or FASTER) Act was signed into law in 2021 and as a result sesame was added to the list of major food allergens requiring mandatory labeling. The law was clearly designed to protect those patients affected with sesame allergy. However, in an epic twist, some food companies have chosen to add small amounts of sesame flour (8) to products that were previously sesame-free to reduce their own liability (instead of conducting the cleaning required to ensure the foods are without sesame). So instead of making products safer for patients with sesame allergy, it may have had the opposite effect.

This is all to say, progress is hard even with the best intentions. As with Newton's third law, every action has an equal and opposite reaction. So while we push forward toward the ultimate goal of a cure, we cannot lose sight for whom we fight. Our efforts must value the patient's experience and be wary of unintended consequences—in the risk, burden, expense and psychosocial effect they may have on our patients.
